# Power Amplification for Jumping Soft Robots Actuated by Artificial Muscles

**DOI:** 10.3389/frobt.2022.844282

**Published:** 2022-03-03

**Authors:** Adriane Fernandes Minori, Saurabh Jadhav, Haojin Chen, Samantha Fong, Michael T. Tolley

**Affiliations:** ^1^ Department of Mechanical and Aerospace Engineering, University of California, San Diego, CA, United States; ^2^ School of Computer Science, Human and Computer Interaction Institute, Carnegie Mellon University, Pittsburgh, PA, United States

**Keywords:** soft robot, jumping, liquid crystal elastomer, power amplification, modular system design

## Abstract

Robots composed of soft materials can passively adapt to constrained environments and mitigate damage due to impact. Given these features, jumping has been explored as a mode of locomotion for soft robots. However, for mesoscale jumping robots, lightweight and compact actuation are required. Previous work focused on systems powered by fluids, combustion, smart materials, electromagnetic, or electrostatic motors, which require one or more of the following: large rigid components, external power supplies, components of specific, pre-defined sizes, or fast actuation. In this work, we propose an approach to design and fabricate an electrically powered soft amplification mechanism to enable untethered mesoscale systems with continuously tunable performance. We used the tunable geometry of a liquid crystal elastomer actuator, an elastic hemispherical shell, and a pouch motor for active latching to achieve rapid motions for jumping despite the slow contraction rate of the actuator. Our system amplified the power output of the LCE actuator by a factor of 8.12 × 10^3^ with a specific power of 26.4 W/kg and jumped to a height of 55.6 mm (with a 20 g payload). This work enables future explorations for electrically untethered soft systems capable of rapid motions (e.g., jumping).

## 1 Introduction

Soft robots—i.e., robots composed primarily of soft materials—have the potential to be more adaptable, conformable, and resilient than their rigid counterparts (Rus and Tolley, 2015). While many soft robots move slowly using bioinspired locomotions such as crawling, walking (Shepherd et al., 2011; Tolley et al., 2014a) or slithering (Luo et al., 2015), previous work has taken advantage of the properties of soft materials to achieve rapid locomotion, for example jumping. A soft body can help jumping robots absorb the impact of landing without the need for dexterous landing capabilities, enabling researchers to explore the customization enabled by rapid fabrication techniques (Tolley et al., 2014b; Bartlett et al., 2015; Duduta et al., 2019). To achieve mesocale jumping, compact and lightweight actuation is necessary, particularly towards self-contained systems (i.e., untethered with all components on-board). However, previous actuation methods for soft jumping robots used pneumatic actuation (Gorissen et al., 2020), chemical combustion (Tolley et al., 2014b; Bartlett et al., 2015), or high-voltage artificial muscles (Duduta et al., 2019) that required either large rigid components or heavy external power supplies.

Another challenge of soft jumpers is the trade-off between weight and speed, given their dependency on the size of the system and its actuation. Smart materials offer lightweight, variable actuation speeds, and can be triggered by different stimuli (e.g., light, magnetism, moisture) (White et al., 2008; Ahmed, 2015; Gelebart et al., 2017; Kularatne et al., 2017; Davidson et al., 2019). However, these methods are challenging to integrate with other components (e.g., batteries, sensors, controllers) towards self-contained robots. On the other hand, electrically activated systems can be integrated with compact, commercially available or rapidly-fabricated parts for self-contained systems (He et al., 2019a; Zhakypov et al., 2019). Specifically, low-voltage (i.e., < 12 V) and electrically driven smart materials enable self-contained and lightweight systems with compact circuitry, but previously explored methods have been limited when applications require rapid motions such as jumping (Hawkes et al., 2010; Minori et al., 2017; He et al., 2019a). This time response is tied by the composition and synthesis of these smart materials, as well as their source of stimulation for actuation such as temperature, pH, and moisture (Madden et al., 2004; Hager et al., 2015).

In nature, rapid motions can be achieved by fully soft animals and plants such as the gal maggot larvae and the Venus Flytrap, respectively. The former can jump heights comparable to fleas (arthropods), and are thus among the best jumpers (measured as a function of body size) in nature (Farley et al., 2019). The gal maggot larvae uses a latch-mediated spring-actuated mechanism that is slowly but forcefully actuated for jumping (Farley et al., 2019; Longo et al., 2019). This larvae jumps by forming an loop with it is body, with its head and tail held together by an adhesive latch, while it contracts its muscles to store elastic energy; when unlatched, this energy is released, as the larva pushes against the ground and takes off. Venus Flytraps, by contrast, are able to generate ballistic motions to trap insects for nutrition due to snap-buckling instabilities in their body (Forterre et al., 2005). Previous work has emulated these biological motions using artificial muscles (Ahn et al., 2019; Guo et al., 2021), or fluidically actuated shells (Gorissen et al., 2020). However, these approaches are challenging to adapt for mesoscale self-contained jumping, as aforementioned.

Analogous to gal magots and Venus flytraps strategies for rapid motions, a common strategy to amplify the power output from an actuator in robots is to use an amplifying system/mechanism (e.g., a motor-spring-latch system) (Ilton et al., 2018; Longo et al., 2019). Particularly, the advantage of using a motor-spring-latch system for the power output of the actuator is that it allows spatial and temporal separation between each element, simplifying the synthesis and analysis of various designs. This type of system also enables the usage of artificial muscles that, while lightweight, have slow actuation speed but with sufficient force to transfer energy to the spring for jumping (Chen et al., 2018; Ilton et al., 2018).

Liquid Crystal Elastomer (LCE) is an example of an artificial muscle that is soft, can reversibly deform given a stimulus (e.g., temperature), enables untethered systems, has good work density (150 kJ/m^3^), and offers tunability in stiffness and actuation strain (via chemical synthesis), as well as geometry, and the direction of deformation (Ahn et al., 2019; He et al., 2019b; Minori et al., 2020). LCE contains internal molecules (mesogens) that depending on their orientation, they will dictate the direction of the deformation of the elastomer when it is activated by a stimulus (e.g., temperature) (Ohm et al., 2012). This feature and patternability of LCE in fabrication and design has not been explored within a system for the design of rapid locomotion in robots, potentially because of the low actuation speed of this artificial muscle (He et al., 2019b; Minori et al., 2020).

Artificial muscles made of LCE also have similar properties to skeletal muscles of mammals (Thomsen et al., 2001; Tian et al., 2018); in particular, during contraction, the maximum force that these muscles can exert decreases due to their length-tension relationship (Gordon et al., 1966; Rosario et al., 2016). This specific behavior in force-displacement can limit the performance of the actuator for loading a linear spring since the total stored elastic energy is proportional to force applied at the maximum displacement (Rosario et al., 2016). Previous work focusing on energy storage for jumping has demonstrated that nonlinear springs with variable stiffness (i.e., stiff to soft) can store more energy than linear springs (Sadeghi et al., 2018; Sadeghi et al., 2021).

Shells are an example of snap-through mechanisms (i.e., when the equilibrium of a system suddenly ceases to exist or becomes unstable when a control parameter, as load, varies, rapidly changing between states (Arena et al., 2017; Gomez et al., 2017)); and can function as powerful nonlinear springs, offering fast output and energy release to trigger rapid motions. Furthermore, the force-displacement characteristics of these nonlinear springs can be easily customized and rapidly fabricated by adjusting their geometry and material parameters (Bende et al., 2015; Chen et al., 2018; Kim et al., 2021). Previous work has demonstrated the impact of the geometry and material of a shell on its stability (Madhukar et al., 2014; Arena et al., 2017), which can be classified as monostable (the shell re-inverts to its original state, or spontaneously snaps-back once the applied load is removed); pseudo-bistable (it has a small delay before spontaneously re-inverting); and bistable (the shell does not snap back when the initial applied load is removed, and it requires another load in the opposite direction to re-invert the shell). The disadvantage of bistable shells is that they require actuation, physical work, to reach each stable state (i.e., to snapped-through/inverted and snapped-back/re-inverted), thus requiring another actuator, adding weight and design requirements or complexity to the system.

**FIGURE 1:**
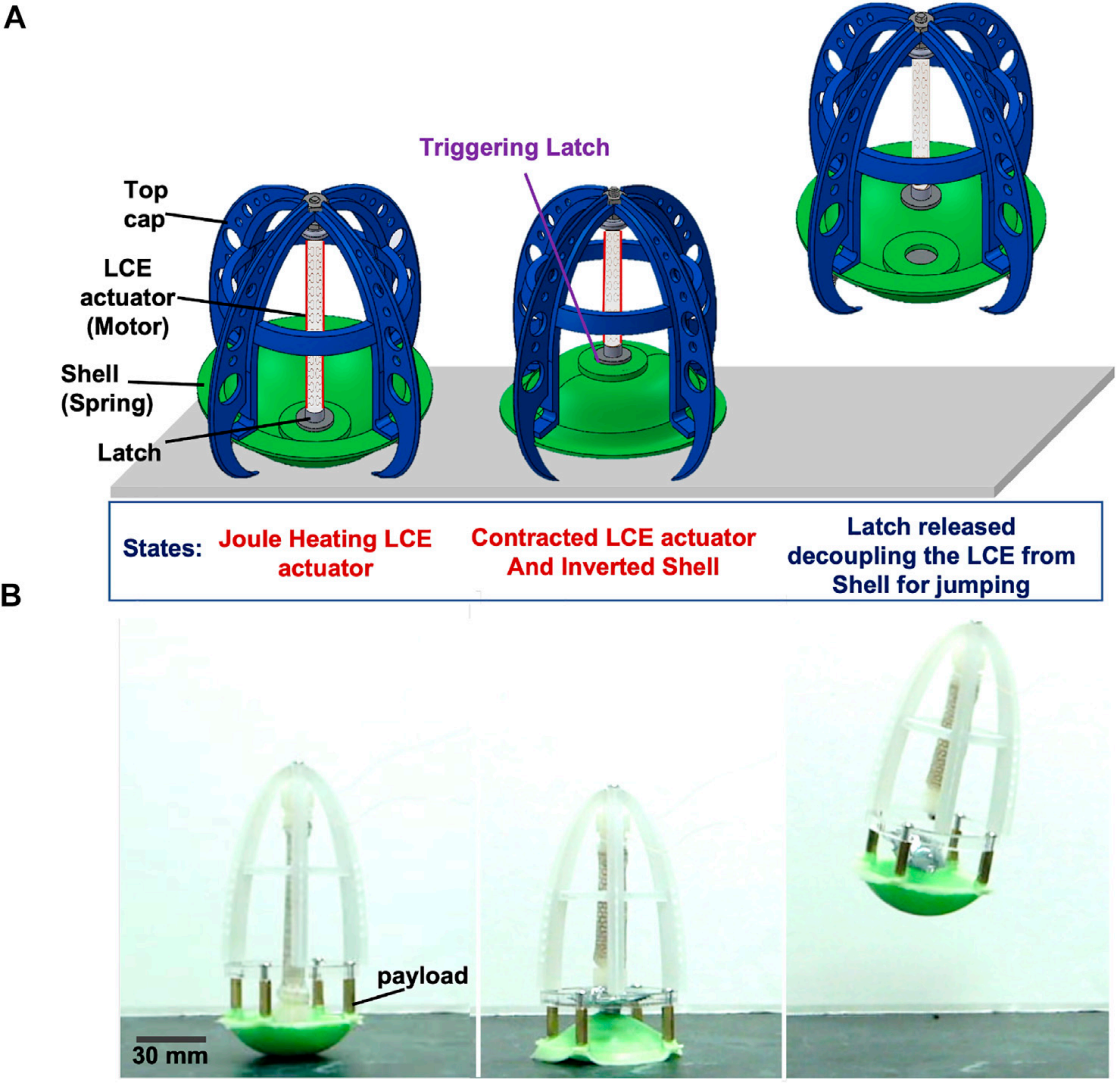
Soft jumping robot with the soft-power amplification system. The basic design consists of an actuator (with one side constrained by a stiff part, such as the top cap) that slowly compresses a spring (i.e., inverts an elastic shell), and a latch that quickly releases the stored energy for jumping: **(A)** Conceptual schematic indicating the states of the soft robot for jumping using the soft amplification system: initial state starting the actuation of the LCE muscle (left); upon activation of the muscle by joule heating, it contracts, inverting the shell (middle); when the latch is triggered, the muscle decouples from the shell, allowing the latter to snap-back to the ground and propel the robot into the air. **(B)** Images from experiments depicting the steps described in **(A)** of the implemented design concept, in the same order, with added payload (with mass of 20 g).

In this work, we focus on the challenge of power amplification of soft actuators for jumping while enabling system integration towards self-contained machines. We propose an approach to the design and fabrication of a modular soft robot with a mechanism for amplifying the power output of artificial muscles to achieve rapid motions (i.e., jumping) using a motor-spring-latch design (Figure 1). To achieve this design with soft components that can be customized and rapidly fabricated, we had to address the challenge of the slow actuation rates of soft artificial muscle actuators such as those electrically actuated like LCE. To solve this challenge, we coupled our soft muscle to a spontaneous snap-back elastic shell, and by decoupling the two with a latch triggered by a soft pouch, we were able to tune the energy stored for jumping while minimizing the requirements on the muscle (e.g., force, length, speed). Furthermore, the continuous adjustability of each of these components (as opposed to the discrete options available for typical robotic systems) allowed us to tailor their capabilities to tune the performance of the entire system for jumping.

## 2 Design Principles and Fabrication

**FIGURE 2:**
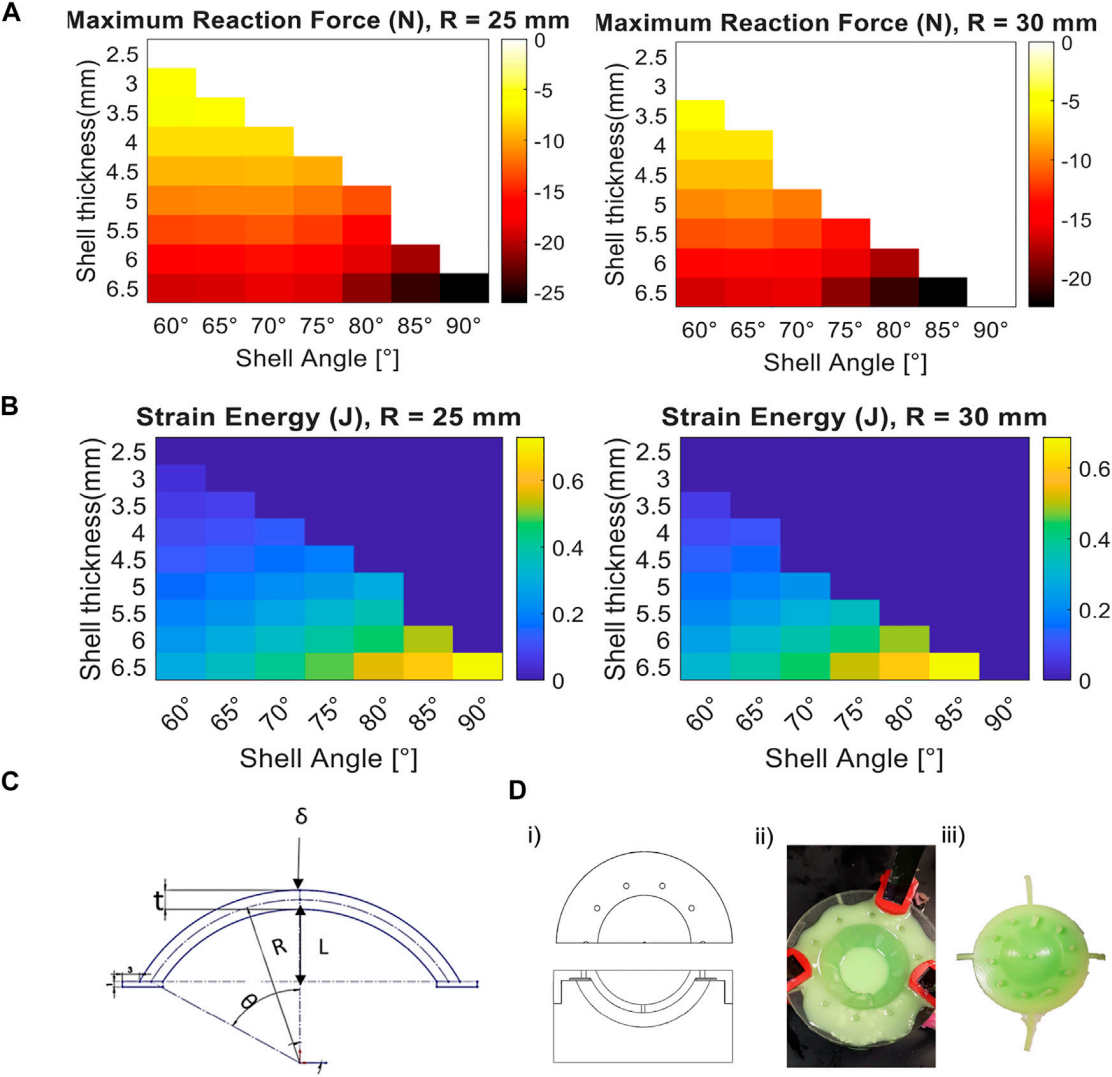
Design requirements for energy storage in snap-backing shells and their fabrication process. **(A)** Heat map from simulation of the maximum force required to invert the snap-backing shells, for various shell geometries (white regions indicate the corresponding shells that do not self-snap-back). **(B)** Heat map of the strain energy of the shells in **(A)** when their geometry varies (dark blue regions indicate the corresponding shells that do not self-snap-back). **(C)** Schematic of the tunable parameters used in the simulation, where *R* is the radius of the shell, fixed per design space explored, *δ* is the prescribed displacement, *L* is the vertical distance from the symmetric center of the shell to half of its thickness (*t*), and *θ* is the angle of curvature of the shell. **(D)** Casting process for the fabrication of the shell, from left to right: **(i)** top and side view of the schematic of the mold used for casting (left), **(ii)** clamped mold inserted into the pressure chamber (middle) containing silicone-based material, **(iii)** and removed part with remaining barbs.

**FIGURE 3:**
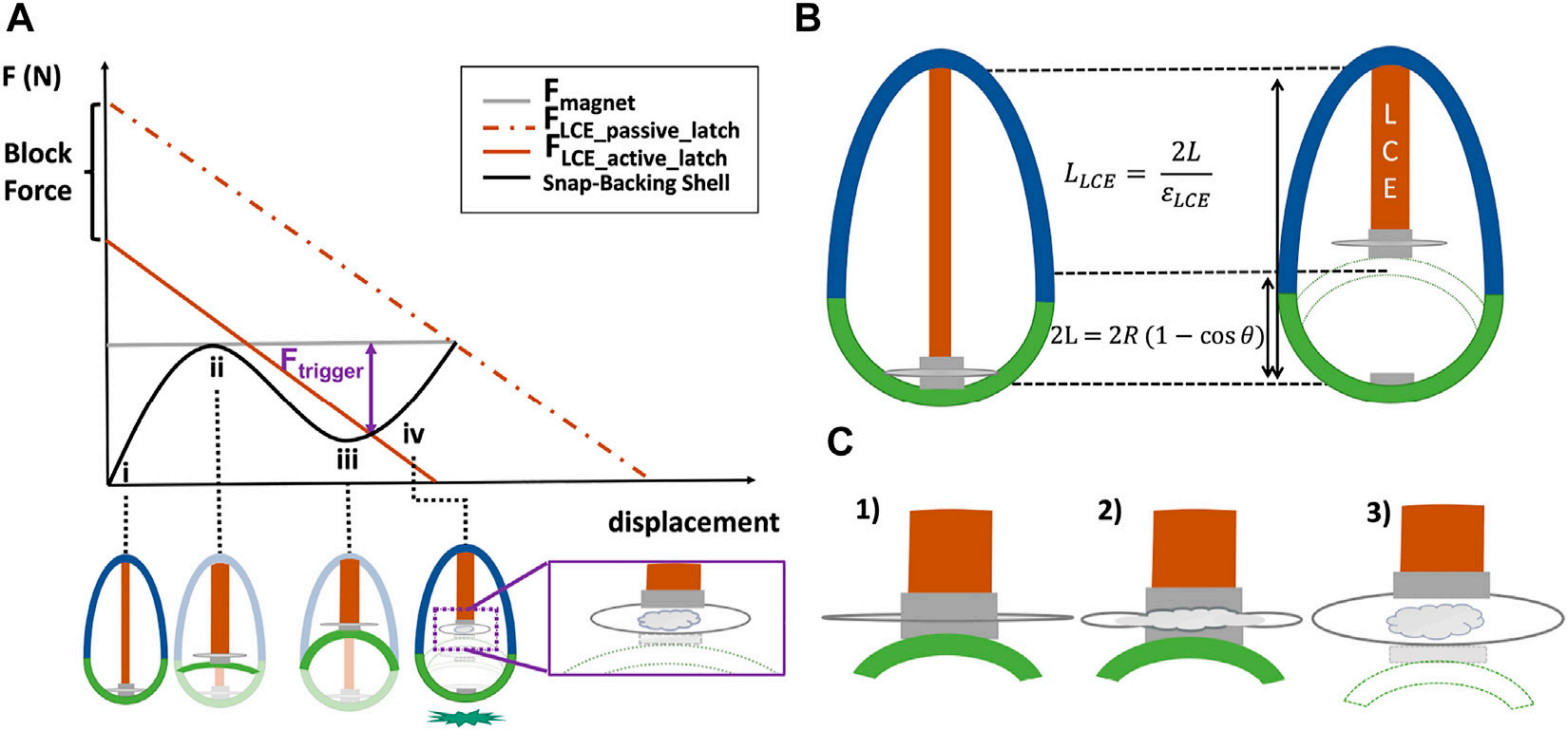
Continuously variable design parameters enable the selection of an actuator, elastic shell, and latch that can be tuned to maximize energy storage. **(A)** Representative force-displacement plot for the elastic shell and of the LCE muscle. **(i)** represents the initial configuration of the shell (non-inverted); **(ii)** its marginal inversion position; **(iii)** the snap-through (inverted) state, and **(iv)** trigger point past the snap-through region. **(B)** Kinematic constraints on the top cap length as a function of the geometry of the LCE and the shell. **(C)** Schematic of the behavior of the active latching when triggered. From left to right, 1) the sandwiched pouch between two magnets (one connected to the LCE and the other to the shell); 2) the low-boiling point liquid starts to evaporate and inflate the pouch; then, 3) when the *F*_*trigger*_ is enough to detach the two magnets (i.e., minimum gap is created), the shell is free to snap-back to the ground.

Our proposed design uses a motor-spring-latch system composed primarily of soft components, where the LCE actuator functions as the motor, the elastic shell as a nonlinear spring, and the latch as a triggering mechanism to quickly disconnect the shell from the actuator (Figures 1A,B). Once the LCE is joule heated and contracted enough to invert the shell, the latch is subsequently triggered, to allow the shell to snap-back and release the energy stored for jumping. A key advantage of this approach is that the continuously variable design parameters of the three main components can be chosen to tune the performance of the system for jumping (i.e., to select a shell, Figure 2, and latch ideally suited for a given actuator, Figure 3A). The nonlinear stiffness curve of the shell enables the system to capture more of the area beneath the force-displacement curve of the actuator to maximize the work done to the shell by the actuator (i.e., area under the shell, $\int_{0}^{x}F\left(u\right)\;du$ , where *x* is the desired displacement of the shell, F the force, and *u* the displacement in the integral) than if compared to the area limited by the constant stiffness of a linear spring (i.e., *kx* ^2^/2, where *k* is the stiffness of the spring and *x* its desired displacement).

To manufacture iterations of these components with various capabilities, we took advantage of rapid fabrication approaches, including molding, laser cutting, and printing.

### 2.1 Design and Fabrication of the Shell for Optimal Energy Storage and Release

To identify the impact of the geometry of the shell (i.e., radius, thickness, and angle of curvature) on the shell’s stability, the strain energy stored in the shell when inverted, and the force required to invert the shell, we used Finite Element Analysis (FEA, Ansys Mechanical APDL), (Figure 2A). The identification of the stability and dynamics of thick hemispherical shells is an active area of research given the complexity and non-linearity of the dynamics during snap-through and snap-back (Gomez et al., 2019). While there has been significant analytical work related to snap-through, most of these studies have focused on thin shells (i.e., t ≪ R), whereas for thick shells, numerical simulations have been necessary (Santer, 2010; Brinkmeyer et al., 2012; Madhukar et al., 2014; Gorissen et al., 2020).

For simplicity and given the relatively slow actuation of the LCE and inversion of the shell, we assumed a quasi-static motion in simulating the snap-through motion of the shell (Gorissen et al., 2020). For the dynamics of the system beginning at the moment of take-off (i.e., to predict the height of the robot over time during a jump), we used a lumped parameter model (see Supplementary Material: Lumped Parameter Model for Jumping Height over Time with a Snap-through Shell). As in previous work (Madhukar et al., 2014), we assumed that the shell can rotate and displace horizontally on its edges (i.e., we assumed roller constraints) and prescribed a displacement to the apex of the shell to estimate the reaction force and energy stored in the shells. We used the Arruba-Boyce material model with an initial shear modulus (*μ*) of 3.5 MPa and an incompressibility parameter value of zero. To simulate the cross-section of the shell, we used eight-node axisymmetric quadrilateral elements.

In simulation, we identified the desired regime of design parameters for snap-backing shells (i.e., the parameters such as geometry and reaction force for which the shell would spontaneously revert when inverted) and the energy stored for each set of viable design parameters (Figures 2A–C). For a mesocale robot (i.e., height and length < 15 cm), we investigated shells with a radius in the range of 25–30 mm radius, thickness in the range 2.5–6.5 mm, and angle of curvature in the range 60–90°.

For the set of design parameters that we investigated, the shell that stored the maximum energy had a radius of 25 mm, since higher angles led to bistability (Figure 2B). However, the trade-off for high energy storage was the force required from the actuator to invert the shell (Figure 2A). Since higher actuator forces required larger actuators (and thus larger systems), even though a particular shell could store more energy, it was not necessarily ideal when considering the constraints it placed on the rest of the system (e.g., on the actuator and the latch).

To fabricate these shells, we used silicone (Elite Double 32, Zhermack, Inc.) and standard casting process for soft devices (Rus and Tolley, 2015; Gorissen et al., 2020). We 3D printed the molds (out of VeroClear material on an Object Connex 500, Stratasys Inc., see schematic of the mold in Figures 2Di), added a release coating layer and waited for it to dry as recommended by the manufacturer (ease release 200 spray, Smooth-On, Inc.). After mixing the silicone using a centrifugal mixer (for 1 minute), we poured the solution into the molds. Later, we clamped the molds to guarantee uniform closure and a consistent shape (Figures 2Dii). Finally, we let the solution cure (15–20 min) inside a pressure chamber (at 40 psi, 0.276 MPa) to minimize bubbles in the cured sample, and later remove the shell out of the mold (Figures 2Diii).

### 2.2 Design Requirements and Fabrication for Actuation and Latching

In this section, we describe the methodology we used to tune the geometry of the LCE, as well as the relationship of the LCE muscle with the shell and latching system. Depending on the size of the system and the desired jumping height, the relationship between the parameters of the LCE, shell and latch can be used to solve for the required parameter values (Figure 3). For instance, the actuator needs to provide enough force and contraction to invert the shell (Figures 3Ai,ii) past its snap-through position (Figures 3Aiii–iv). The latching system needs to hold enough load during the inversion of the shell and when its inverted (Figure 3A), and to trigger quickly to enable jumping.

#### 2.2.1 Tunable Actuation

Achieving the force and contraction necessary to invert the self-snapping back shell required adjusting the cross-sectional area of the actuator and its length, respectively (Figure 3B). To obtain an overall mesocale jumping system (i.e., length and width ≤15 cm), the size of actuator and latch as well as the weight of the system are also constraints. For instance, the geometry of the LCE muscle and its maximum actuation strain (i.e. at no-load) are variables necessary to consider for designing the top cap (stiff component; Figure 3B). The height of the top cap is a function of the geometry of the shell (radius, theta) and the contraction of the LCE muscle when heated. Thus, the height of the top cap can also be assigned as a design constraint since the weight of the top cap increases with the geometry of the LCE and shell, and overall increasing the size required for a mesoscale jumping robot. Thick (i.e., > 1.5 mm) and long actuators (i.e., > 15 cm) actuators would also not fit onboard a small scale jumper, motivating the additional constraint of height and length < 15 cm.

**FIGURE 4:**
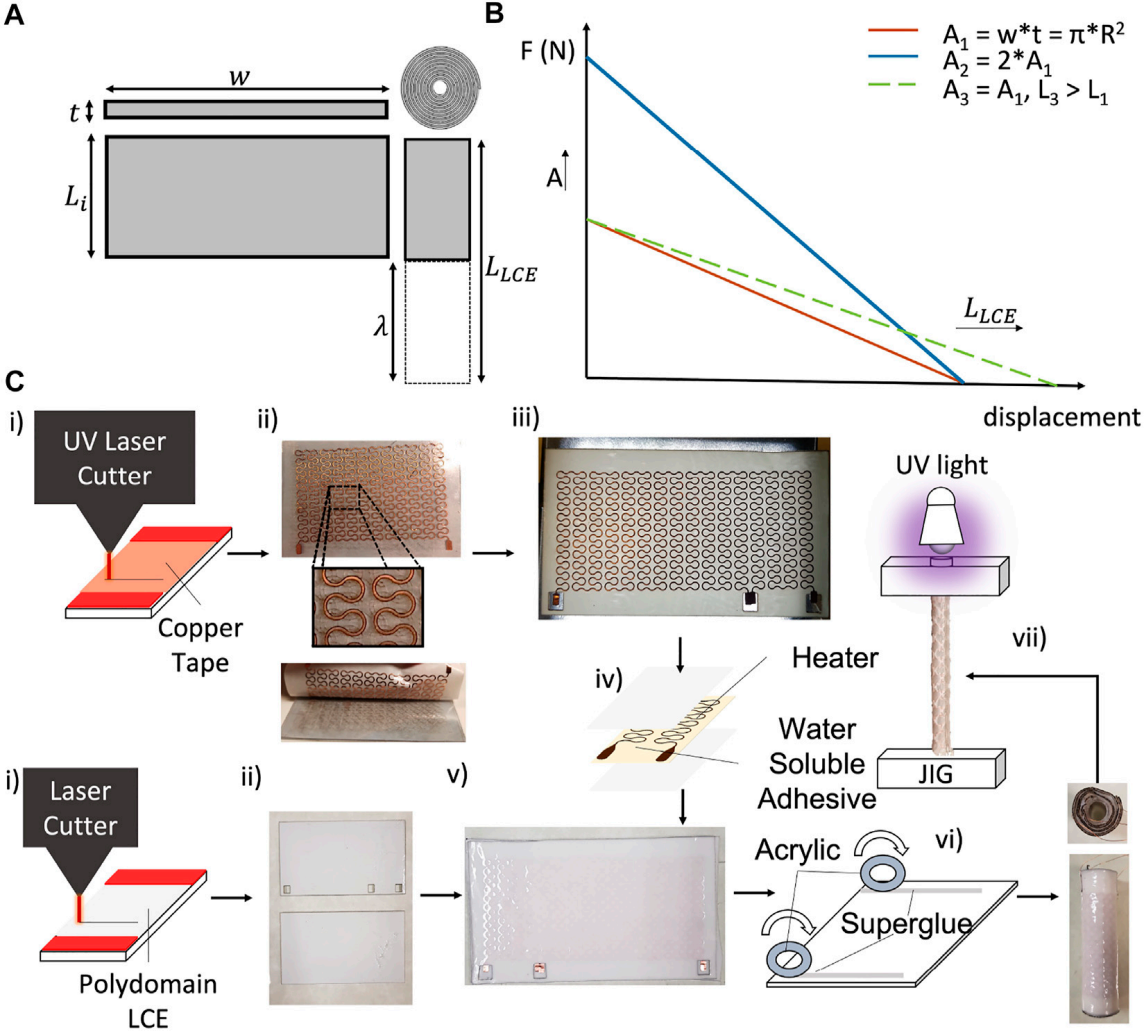
Design guidelines and fabrication process for rolled LCE actuators. **(A)** Design parameters used to tune the geometry of the LCE, where *w* is width, *t* initial thickness. **(B)** Representative plot indicating the tunability of the rolled actuator force-displacement characteristic, based on the dimensions of the initial LCE sheet, where *A*_*i*_ is the cross-section area, and *L*_1_, *L*_2_, *L*_3_ are the lengths of the actuator. *Y*-axis intercepts correspond to the blocked force of each actuator, and *X*-axis intercepts correspond to their corresponding no-load displacements. **(C)** Process of fabrication of the rolled actuator (from left to right as indicated by arrows): parallel patterning of the heaters and LCE sheets (with UV and CO_2_ laser cutters, respectively), followed by the transferring process of the circuit onto the LCE sheets, to finally roll and stretch the LCE actuator for alignment.

To satisfy these constraints on the size and actuation performance of the muscle, we developed an approach to design and fabricate rolled LCE muscles that enabled us to continuously adjust their capabilities by taking advantage of the patternability possible when using LCE synthesis (Figure 4). First, we chose to align the mesogens of the LCE with mechanical strain. To achieve this, we stretched a sheet of partially (loosely) cross-linked LCE. Once the sheet was later cured with ultravioulet (UV) light, the result was an actuator with the ability for uniaxial actuation when heated. This synthesis process (Yakacki et al., 2015) allowed for the fabrication of patternable sheets via casting (Minori et al., 2017; He et al., 2019a; Minori et al., 2020) (see Supplementary Material for more details on the LCE synthesis). Prior to curing with UV light, we took advantage of the compliance of the LCE and rolled the sheets into a tubular shape. The required initial dimensions of the LCE sheets before stretch (i.e., before alignment, Figure 4A) were estimated assuming an incompressible neo-hookean material under uniaxial stretch where: *λ* is defined as the ratio between final stretched length of the LCE (*L*_*LCE*_) over *L*_*i*_, its initial length before stretching the actuator. To obtain an estimate for the maximum force of the stretched actuator with specified dimensions and material properties at a given displacement, we assumed a linear relationship between force and displacement (Figure 4B). These estimations were based on the characterization of the performance of the LCE actuator (i.e., measured blocked actuation force and no-load strain of the aligned actuator).

As discussed above, completion of the two-step polymerization process required UV curing of the strained actuator, however, we first needed to add an electrical heating layer to later control the activation of the cured muscle. For the heating layer, we used a 0.035 mm thick copper tape attached to an adhesive carrier (Gel-Pak, delphon) and defined the serpentine shape of the heater traces (∼5 Ω, 0.42 mm wide), which was cut using a custom UV micromachining system (∼10 *μ*m spot size (Chopra, 2018)), see Figures 4Ci, top. Once patterned, we removed the copper layer from the adhesive carrier (Figures 4C,ii, top) and used a water soluble tape to transfer the copper layer onto the polydomain (loosely-crosslinked) LCE sheet (Figures 4Ciii). After dissolving the tape with deionized water (DI), we then sandwiched the copper layer with another sheet (Figures 4Civ,v) of laser cut polydomain LCE (Figures 4Ci,ii, bottom).

To complete the fabrication of the actuator, we rolled the sandwiched LCE with the patterned heater into a tubular form, mechanically stretched the muscle, and cured it using UV light Figures 4Cvi,vii). To achieve this, we first deposited strips of cyanoacrylate adhesive (*Gorilla* Super Glue, *Gorilla* Glue Co.) along two opposite edges of the LCE sheet, and rolled it up using two acrylic discs as rolling guides to form a tubular LCE actuator (Figures 4Cvi). These discs were also used to guarantee that the inner rolled layers were uniformly constrained when adhered to the end-caps for mechanical stretch (Supplementary Figure S3C). Once stretched (*λ* = 2), the tubular actuator was cured under UV light for an hour. We used a glass rod to transmit the UV light inside of the rolled actuator to promote curing in the interior of the actuator.

#### 2.2.2 Active Latching

For the latch constraints, it was important to achieve a fast release (Ilton et al., 2018) to avoid inefficiency in energy release from the actuator to the shell. Being compact and reversible was also important so the latch did not compromise the weight, or performance of the robot (tied to its geometry, Figures 3A,B). We also required the latch to enable reversible latching, which in turn required the latch to promote self-alignment after jumping to reset the connection between the shell and the artificial muscle.

To achieve a self-aligning latch, we used high-temperature neodymium magnets with sufficient magnetic strength to connect the actuator to the shell while the former inverted the latter. To quickly trigger the decoupling of the actuator from the shell with a compact latch, we used a pouch partially filled with liquid with a low temperature boiling point, inserted between the two magnets (Figures 3Aiv,C). Once the LCE muscle provided enough force and displacement to invert the shell, the liquid in the pouch was heated, causing it to boil and inflate the pouch, peeling the magnets apart to separate the magnets attached to the surface of the pouch (Figures 3A iv–C, 1–3; Supplementary Figure S5). This concept allowed for a lightweight, compact and simple latching system that could be electrically triggered.

The primary reason for using an active latching system was that it reduced the force required from the actuator at a large displacement to separate the magnets (i.e., *F*_*trigger*_ = *F*_*magnet*_ − *F*_*LCEactive*_ _*latch*_, where *F*_*trigger*_ is the triggering force of the latch, *F*_*magnet*_ is the attraction force of the magnets, and *F*_*LCEactive*_ _*latch*_ is the actuation force required by the LCE when using an active latch). The result was a significant reduction in the requirements placed on the actuator. By providing control of when to trigger the shell, this approach minimized the force and length requirements on the actuator (Figure 3Aiv) compared to a system that relied only on a passive latch (i.e., on the force of attraction of the magnets).

To manufacture the pouch, we followed a fabrication process similar to previous work (Narumi et al., 2020), specifically: 1) we used commercially available anti-static circuit bags as the layers for the pouch (25 mm × 25 mm); 2) we cut and sealed the bags together, forming a pouch with a small opening; 3) we injected a commercially available low boiling point liquid with a syringe (acetone, ∼ 80*%* filled); 4) we completed the sealing process with a heat sealer.

## 3 Results and Discussion

### 3.1 Tuning Jumping Heights by Varying the Parameters of Elastic Shells

**FIGURE 5:**
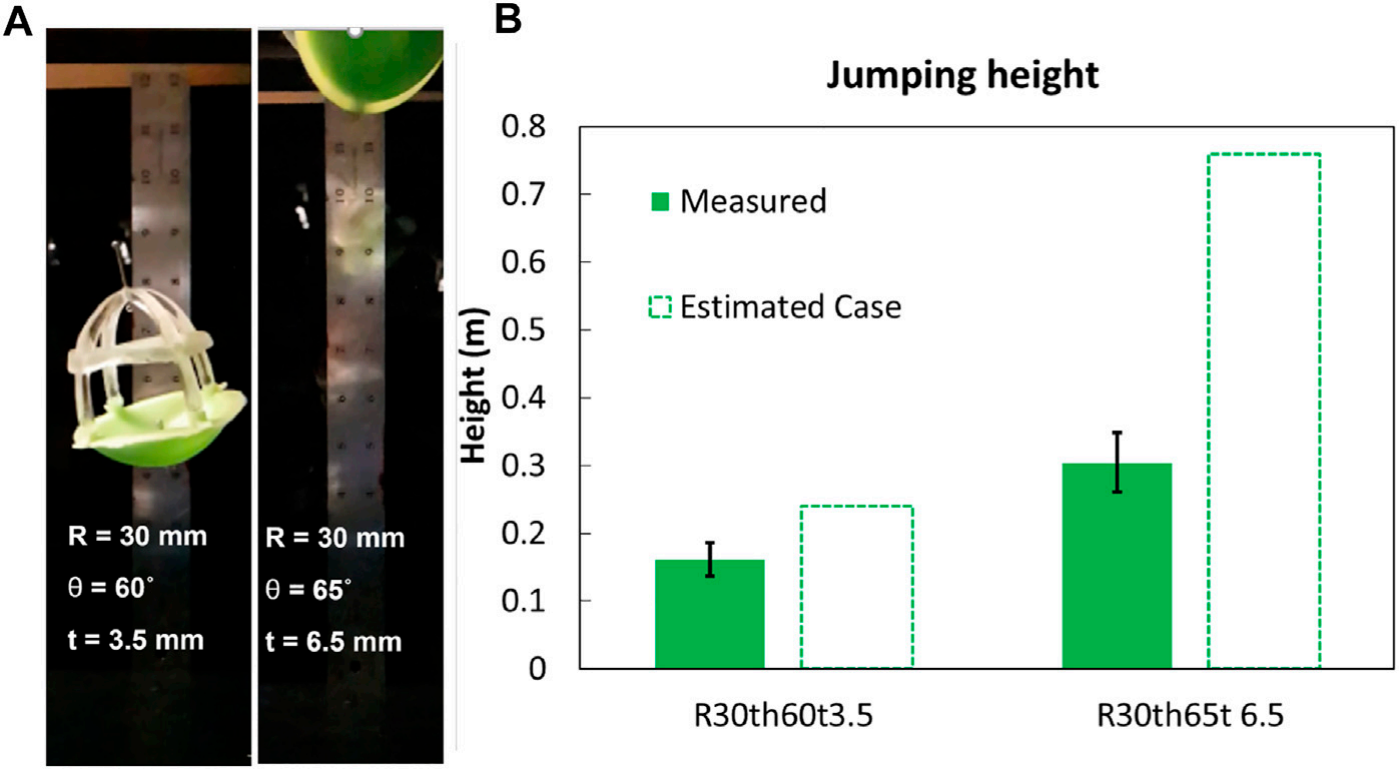
Validation of model of jump height as a function of elastic shell parameters. **(A)** Stills from a high-speed video of the maximum jump height of the devices used to validate the impact of varying the parameters of the elastic shells. **(B)** Comparison between the performance of devices with shells with different geometries and their estimated case, and measured height in three trials.

To validate our simulation of the relationship between the shell dimensions (see Section Shell Design and Fabrication for Energy Storage) and the jumping height of the robot (assuming conservation of energy), we tested two shells designs with the same radius (30 mm) but different thicknesses (3.5 mm, 6.5 mm) and angles of curvature (60°, 65°; see Figure 5). For this experiment, we emulated the behaviors of the artificial muscle and latch with a fishing line connecting the inverted shell to the top cap. To allow the shell to invert, we bonded the base of the top cap to the perimeter of the shell with soft feet (same material as the shell) using a cyanoacrylate-based adhesive set as instructed by the manufacturer (PolyKit, ttbonding Inc., Supplementary Figure S4). We manually inverted the shell and affixed the fishing line to the top cap with a screw. Once inverted, we triggered the shell using a heat gun to melt the fishing line, simulating the action of the latch. This trigger allowed the snap-back of the shell, causing it to press against the ground, launching the device into the air (see Figure 5A).

The shell with a thickness of 3.5 mm and an angle of 60° resulted in an apical gravitational potential energy (*mgh*, *m* is the total mass, *g* the gravitational constant, and *h* the apical height) and jump height of 35.6 ± 5.4 mJ and 161.7 ± 24.5 mm, respectively (see Figure 5B). The simulated result for the strain energy was 52.9 mJ, assuming the conservative case of stored energy in the shell at its snap-through region, (i.e., Figure 3Aiii, minimum work required to invert the shell), and an estimated height of 239.9 mm. By comparison, the shell with a thickness of 6.5 mm and an angle of 65° achieved an apical potential energy of 109.8 ± 15.9 mJ and jumping height of 304.5 ± 44.1 mm. The simulated results for the strain energy for the best estimated case for this second shell was 274.0 mJ, and an estimated height of 759.8 mm.

These results supported our assumption that, to first order, by changing the geometry of the shell (almost doubling the thickness and slightly increasing the angle) can proportionally impact the energy released and jumping height. The difference observed between the measured and estimated case for the energy stored in each shell (and consequently the jump height of the device), is likely due the small relaxation during the manual locking of the fishing line between the top cap and the shell, as well as the time required to melt the fishing line for the release of the shell for jumping. Despite these effects on the absolute values of the results, the relative values (i.e., the trends) between the simulated and experimental results were consistent, supporting the use of the model to optimize design parameters, and the ability to tune the maximum jump heights by varying the design parameters of the elastic shells. Further, this experiment validated the overall design approach of using a point force (to be generated by an artificial muscle) to invert elastic shells for energy storage, and using the subsequent snap-back for power amplification for a soft jumping robot. The next section will describe the co-design of elastic shells and artificial muscles capable of inverting them.

### 3.2 Actuator Characterization and Shell Selection

**FIGURE 6:**
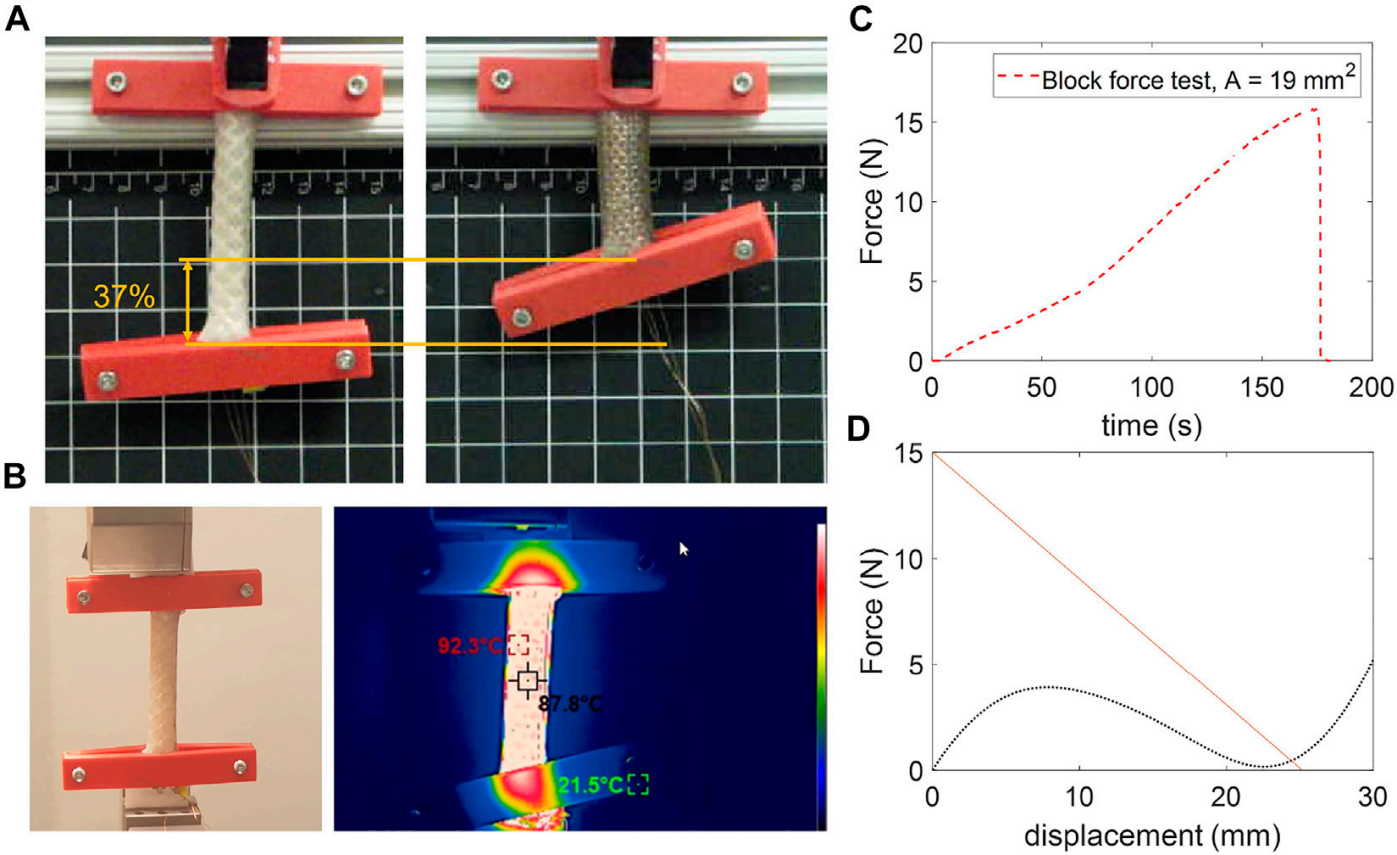
Characterization of the rolled LCE actuator and selection of the shell. **(A)** Actuation strain of the rolled LCE actuator with original length of 68 mm and final length of 43 mm (the flat, pre-rolled dimensions of each sheet were: 72 mm × 52 mm x 1.25 mm). **(B)** Block force setup, and thermal image of the LCE at the point of failure. **(C)** Maximum force measured from the 68 mm long sample with outer diameter of ∼ 10 mm. **(D)** Force-displacement curve of the LCE actuator (orange line) based on empirical data from blocked force and strain, and of the simulation of the shell (dotted black curve).

Given the dependence of the height of the top cap on the length of the actuator, we constrained the maximum length for a single LCE actuator to be ≤75 mm to limit the overall height of the robot. To measure the maximum force generated by the LCE actuator (i.e., the blocked force; actuator cross-sectional area: 19 mm^2^), we affixed the actuator edges with 3D printed parts to a load cell (250 N range, Mark-10 Corp.), then we powered our system (7.3 V, 1.3 A from a constant-voltage power supply) until failure (Figure 6B). We observed a maximum blocked force of 15.8 N before failure by delamination from the clamps at approximately 92 °C (Figures 6B,C) measured with a thermal camera (HT-H8, Hti-Xintai).

To measure the maximum actuation strain of the muscle, we fixed one side of the actuator and allowed the other to contract and displace freely when Joule heated, until ∼ 110 °C. The maximum actuation strain we measured for this sample was about 37*%* (Figure 6A). These results are comparable to previous work that used a similar approach to the synthesis of LCE but unexplored tunability in size of the actuators (i.e., without rolling the layers (He et al., 2019b);). Based on these results and our proposed actuator design, we can use the actuation stress of the actuator to estimate the necessary geometry and force-displacement relationship of our actuator (see Figure 4B, and Section 2.2).

The estimated relationships between the system components (i.e., the actuator, shell, and latch) allowed us to select shells with properties tuned to the capabilities of a given actuator and latch (Figure 3). From the simulated results for the shell performance (Figures 2A,B), we can see that the shell with 30 mm radius, 3.5 mm thickness and 60° angle can be actuated with the LCE muscle described above and an active latching system with a trigger force of 4 N (Figure 6D) without compromising our design constraints in size. Furthermore, this result and design choice still enabled sufficient energy for jumping to emulate minimum payloads for untethered jumping (Figure 5C). This result also highlights the advantage of tuning other parameters such as shell and active latch to minimize the performance requirements on the actuator (i.e., the requirements on the force-displacement relationship), consequently minimizing the size of the actuator.

### 3.3 Soft Power Amplification System Demonstration and Performance

**FIGURE 7:**
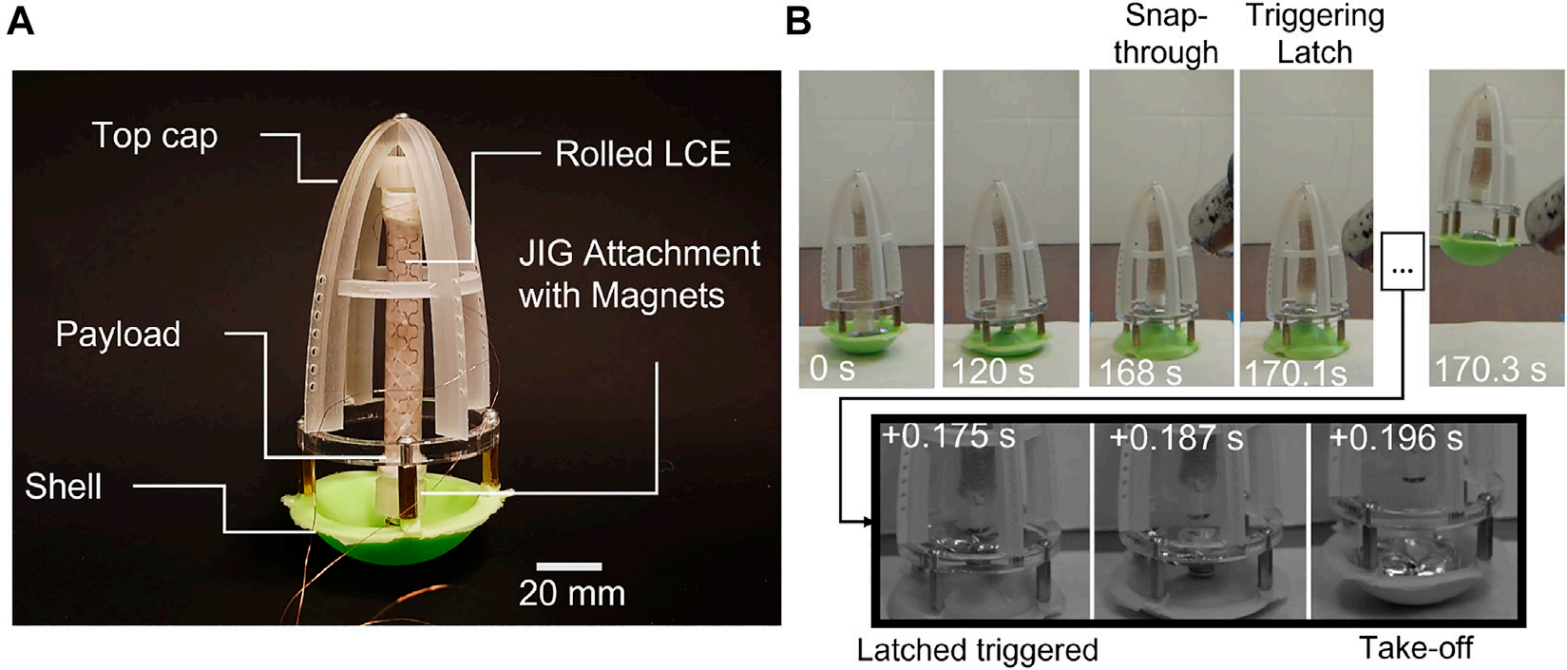
Demonstration of the soft power amplification mechanism for a jumping robot. **(A)** Image of experimental system with key components labeled. **(B)** Top: Frames from video of the LCE muscle inverting the elastic shell until snap-through occurred (0–168 s). Bottom: Frames from high-speed video of the triggering of the latching system, showing the rapid snap-back of the shell once it was decoupled from the LCE muscle.

Based on our design guidelines, we used our power amplification system to build a tethered jumping soft robot (Figure 7A). To simulate a case with payload (with mass of ∼20 g, approximately the mass of two small 3.7 V, 1,300 mA h lithium polymer batteries), we added a modular component made of acrylic attached with stand-offs between the top cap and flexible feet on the shell (labeled as part of the “payload” in Figure 7A). The top cap and acrylic were attached with double-sided adhesive tape (VHB, 3M Corporation), so if necessary, could be easily replaced.

To validate our design concept, we powered the rolled LCE actuator (∼ 7.3 V) until inverting the shell, at which point we heated the vapor activated pouch with a heat gun to separate the connecting magnets between the shell and actuator (Figure 7B). The triggering of the latching system and the resulting rapid motion of the snap-back of the shell were recorded with a high-speed camera (Phantom camera, VEO-410L, Vision Research Inc.).

Once inflated, the pouch successfully decoupled the LCE and the shell, and allowed the latter to snap back to its original shape and propel the robot into the air (Movie S1) to a maximum height of 55.6 mm at 0.49 m/s (average speed from take-off to maximum height) and potential energy (*mgh*) of 35.7 mJ with a total mass of 65.3 g. This total system mass is five times the weight of the elastic shell (13 g).

Assuming the conservative case of no energy losses (i.e., *P* = *mgh*/*t*, where *P* is power, and *t* is time), the work output of the LCE muscle as it inverted the shell was done at an average mechanical power of 0.2 mW over 168.1 s. Whereas with the average power output of the shell during jumping was 1.7 W over 20.7 ms (measured from the triggered detachment of the latch to the take off time of the robot). Thus, the power amplification ratio of our system was 8.12 × 10^3^ and the energy efficiency was 67*%* (comparing the simulated strain energy stored in the shell right before triggering the latch, 53.2 mJ, with the final potential energy of the system at the apex of the jump). This amplification of the power output of the LCE muscle enabled the robot to achieve rapid motion without compromising the advantages of the patternable and continuously variable dimensions of the LCE based soft actuator.

Our modular and soft power amplification system also generated a specific power of 26.4 W/kg, which is higher than values estimated in previous work that used other soft or joule heating actuation such as electrically powered dielectric elastomers (Duduta et al., 2019) or shape memory alloy for actuation (Zhakypov et al., 2019), respectively. These types of actuated systems had their latch and actuator coupled during the triggering of the latch for jumping. Thus, by decoupling the slow activated actuator from the elastic component, our results also indicate an advantage of using this design strategy–to efficiently release the energy stored–than if those components were coupled for rapid motions.

## 4 Conclusion

In this work, we demonstrated an approach to the design of modular soft mechanisms using the snap-through of elastic shells to amplify the power output of soft artificial muscles to achieve rapid motions like jumping. Using this methodology, we can estimate the performance of the system (e.g., jumping height and power amplification) given a set of design parameters for the shell (i.e., radius, thickness, angle of curvature), and actuator (i.e., cross-section area, length) when interacted with an active latching system. With our results, this approach also enables a way to solve the inverse problem (i.e., given a desired jumping height, we can select a shell and actuator design). Additionally, we proposed a new methodology to pattern and fabricate compact, high-force, electrically actuated LCE muscles with continuously variable performance (i.e., in terms of their blocked force and free displacement). Finally, we demonstrated the performance of our soft power amplification system with a mesocale jumping robot.

Our soft jumping robot was able to achieve a jumping height, specific power, and energy conversion efficiency comparable to previous work (Duduta et al., 2019; Zhakypov et al., 2019; Gorissen et al., 2020). These comparisons are based on systems that used soft actuators with similar attempts to enhance the actuation for jumping. To our knowledge, this work is the first to contribute a modular approach that enables continuously tunable performance for jumping and rapid motions using a soft power amplification system.

Future work could further optimize the design of a soft mesoscale jumping robot by investigating: 1) How the methods of synthesizing liquid crystal elastomer for artificial muscles affects the relationships between the performance parameters (e.g., their actuation rate, maximum stress, and maximum strain); 2) on-board electrical control of the latch; and 3) the use of parallel actuators, combined with an optimization of the geometry of the cap, to enhance jumping performance. As an example of 1), there is an ongoing effort to enhance the mechanical properties of LCEs to achieve enhanced actuation performance (Ula et al., 2018). Relating to 2), we used a heat gun to activate and validate our latching system, but it is possible to control the timing of the latch and therefore the jumping height of the robot by integrating a flexible or stretchable circuit onto the pouch (Narumi et al., 2020), without compromising power requirements (5–7 W) for self-contained systems, and pouch performance (e.g., force and temperature) (Nakahara et al., 2017). Similarly, for 3) future work could explore different cap designs optimized to be lightweight or self-righting, or even the use of parallel actuators to invert the shell to store more energy without requiring a longer robot.

Aside from enabling future exploration of untethered mesoscale jumping soft robots, the approach described here may also enable modular, conformable and rapidly fabricated power amplification systems for: i) other mesoscale robotic applications (e.g., swimmers and bioinspired ballistic motions); ii) embodied devices (e.g., mechanical computing); iii) and sustainable systems (e.g., rapidly actuating devices that leverage the inherent intelligence of stimuli driven or biodegradable materials).

## Data Availability

The data generated for this study is available upon request.
