# Supplementary material for: Power Amplification for Jumping Soft Robots Actuated by Artificial Muscles
Source: Front Robot AI. 2022 Mar 3;9:844282. doi: 10.3389/frobt.2022.844282 (PMC8927657; doi:10.3389/frobt.2022.844282)
Supplement: Supplementary file 1 [file DataSheet1.PDF]

Supplementary Material

1 LUMPED PARAMETER MODEL FOR JUMPING HEIGHT OVER TIME WITH A SNAP-THROUGH SHELL

To estimate the jumping height of the robot over time, we used a lumped-parameter model. For simplicity, we used a mass-spring (MS) system that was compressed and released for jumping, equivalent to the configurations of the system immediately and before snapping (Figure S1A, precompressed and take-off).

Coupling our quasi-static Finite Element (FE) simulation with the MS system, we estimated the jumping height by using similar assumptions and steps as in previous work (Gorissen et al., 2020). The stored energy was dependent on the FE simulation results and its assumptions (e.g., vertical reaction force, see *section Shell Design and Fabrication for Energy Storage*). Assumptions for the MS system: i) the system stored an amount of energy equivalent to the numerically simulated energy stored in the shell before snapping-back (i.e., latch is triggered right after snap-through, Figure 3A iv); ii) the spring was initially precompressed by a ΔL_{LCE} (i.e., contraction of the LCE actuator necessary for the assumption above) meaning the lower mass was positioned at a distance $h_{\circ}$; iii) the stiffness of the spring could be approximated as $k = \dfrac{2SE}{\Delta L_{LCE}^2}$, where SE was the strain energy stored in the shell right before snapping-back; iv) the masses of the system were concentrated at the extremes of the system.

The equations of motion for the MS system are Equations (S1, S2), where $L_{\circ}$ is the resting position of the spring (i.e., relaxed state of the LCE actuator), m_1 is the lower mass (i.e., shell, magnet, pouch, and payload), m_2 is the upper mass (i.e., top cap, magnet, LCE actuator) and g = 9.81 m/s^2.

$$m_1\ddot{y}_1 + k(y_1 - y_2) = -m_1 g - kL_o \tag{S1}$$

$$m_2\ddot{y}_2 + k(y_2 - y_1) = -m_2 g + kL_o \tag{S2}$$

We solved the differential equations using the numerical solver ODE45 of MATLAB, in which the coefficient of restitution used was assumed to be 0.5 (Gorissen et al., 2020) and the initial conditions of the system were $[y_{1\circ},\ y_{2\circ},\ \dot{y}_{1\circ},\ \dot{y}_{2\circ}] = [h_{\circ},\ h_{\circ} + L_{LCE},\ 0,\ 0]$. The parameters to solve the equation were m_1 = 0.0327 Kg, m_2 = 0.0325 Kg, $L_{\circ}$ = 0.06654 m, ΔL_{LCE} = 0.024 m (based on the LCE characterization in the section *Actuator Characterization and Shell Selection*), $h_{\circ}$ = 0.015 m and SE = 0.0532 J.

The model predicted a maximum jumping height of 46.2 mm at 0.111 s measured from take-off (0.016 s) while the robot achieved a maximum jumping height of 55.6 mm at 0.113 s measured from take-off (0.021 s) (Figure S1B). Thus, the MS model provided a good prediction for when the events of snapping and maximum height occur over time. For simplicity, we assumed that our model is coupled (i.e., with a constant stiffness before and after snapping with the estimated energy stored right before snapping from FE simulation). Since the actuator was separated from the shell at take-off (i.e., variable stiffness, from stiff to soft behavior when latched, (Sadeghi et al., 2021), we believe this assumption could have contributed to the difference between estimated and measured results. Nonetheless, our results provided a comparable first order approximation to estimate the effect of the system's parameters in its jumping height over time.

2 SYNTHESIS OF THE LOOSELY CROSS-LINKED LCE SHEETS

We followed a two-step polymerization process (He et al. (2019); Minori et al. (2020)). First, we added 10.957 g (18.6 mmol) of 1,4-bis-[4-(3-acryloyloxypropyloxy) benzyloxy]- 2-methylbenzene (RM257, Wilshire company, 95%) into a glass beaker, and added 3.72 g of the toluene, then covered the mixture with aluminum foil to later heat the mixture at 85°C on an oven for 20 minutes. After that, we added 0.0771 g (0.3 mmol) of the crosslinker 2-Hydroxy-4- (2-hydroxyethoxy)-2-methylpropiophenone (HHMP, Sigma-Aldrich, 98%) into the mixture and heated to 85°C again for 10 minutes. Later, while stirring with a magnetic stirring bar, we added 3.076g (16.9 mmol) of 2,2-(ethylenedioxy) diethanethiol (EDDET, Sigma-Aldrich, 95%), the spacer in LCE, the tetra-arm thiol crosslinker, 0.244 g (0.5 mmol) of pentaerythritol tetrakis (3-mercaptopropionate)(PETMP) Sigma-Aldrich, 95%), and 0.038 g (0.4 mmol) of dipropylamine (DPA, Sigma-Aldrich, 98%), the catalyst (dissolved, 1:100, DPA:toluene) into the solution. After stirring the solution for 3 minutes and degassing for 5 minutes, we poured it into the rectangular mold (which allowed for tunable thickness). After 24 hours at room temperature in a dark environment, we placed the mold into the oven for 12 hours at 85°C to achieve the loosely cross-linked polydomain LCE sheets.

REFERENCES

Gorissen, B., Melancon, D., Vasios, N., Torbati, M., and Bertoldi, K. (2020). Inflatable soft jumper inspired by shell snapping. *Science Robotics* 5

He, Q., Wang, Z., Wang, Y., Minori, A., Tolley, M. T., and Cai, S. (2019). Electrically controlled liquid crystal elastomer–based soft tubular actuator with multimodal actuation. *Science Advances* 5, eaax5746

Minori, A. F., He, Q., Glick, P. E., Adibnazari, I., Stopol, A., Cai, S., et al. (2020). Reversible actuation for self-folding modular machines using liquid crystal elastomer. *Smart Materials and Structures* 29, 105003

Sadeghi, S., Allison, S. R., Bestill, B., and Li, S. (2021). Tmp origami jumping mechanism with nonlinear stiffness. *Smart Materials and Structures* 30, 065002

3 SUPPLEMENTARY VIDEO

Movie S1 link: Demonstration of the soft power amplifcation system for a jumping robot.

4 SUPPLEMENTARY FIGURES

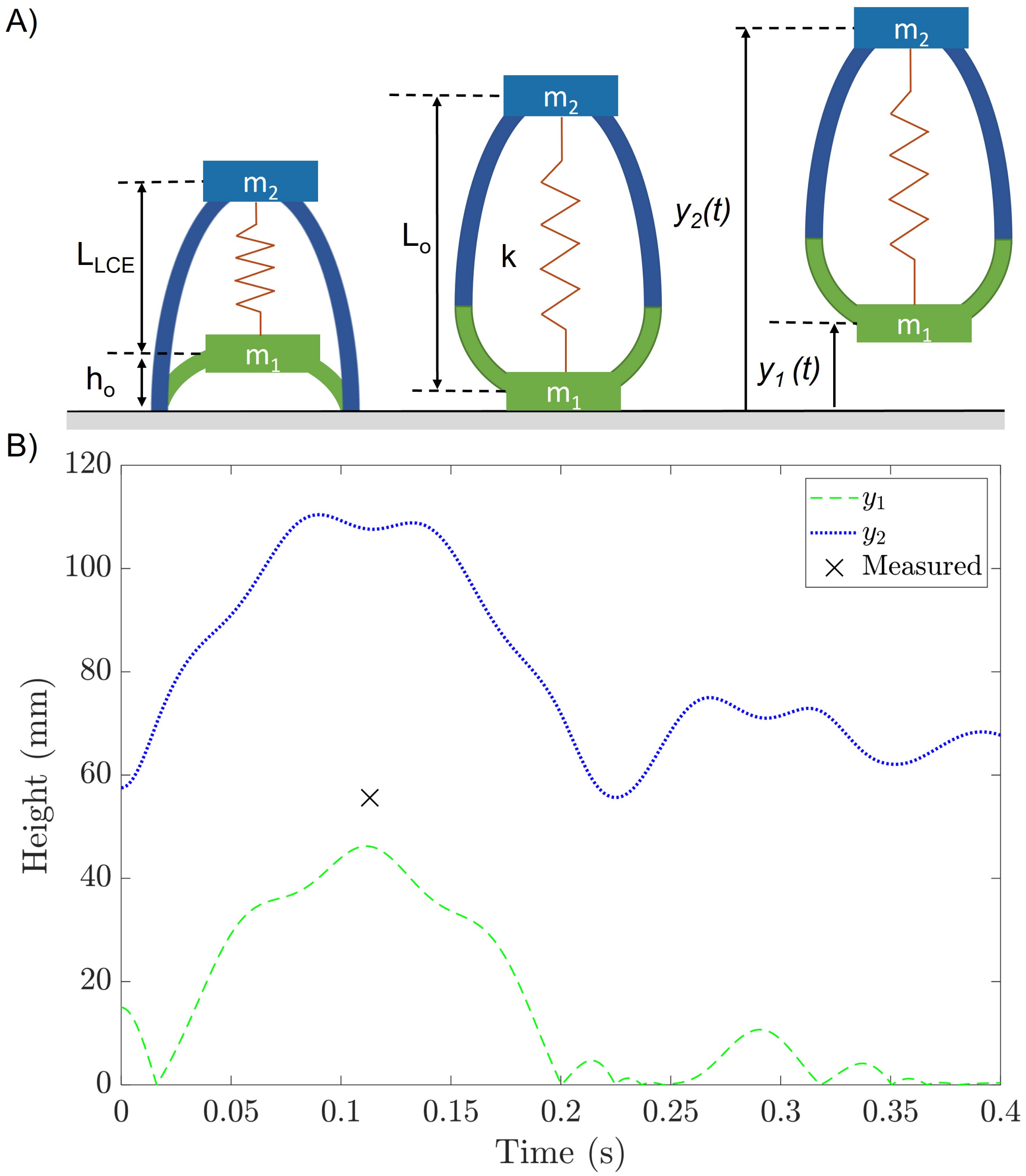

Figure S1. Lumped parameter model for estimation of the jumping height of the robot over time. A) Schematic of the MS system with its respective initial precompressed, take-off, and airborne states. B) Prediction of the jumping height compared to the value experimentally measured (X).

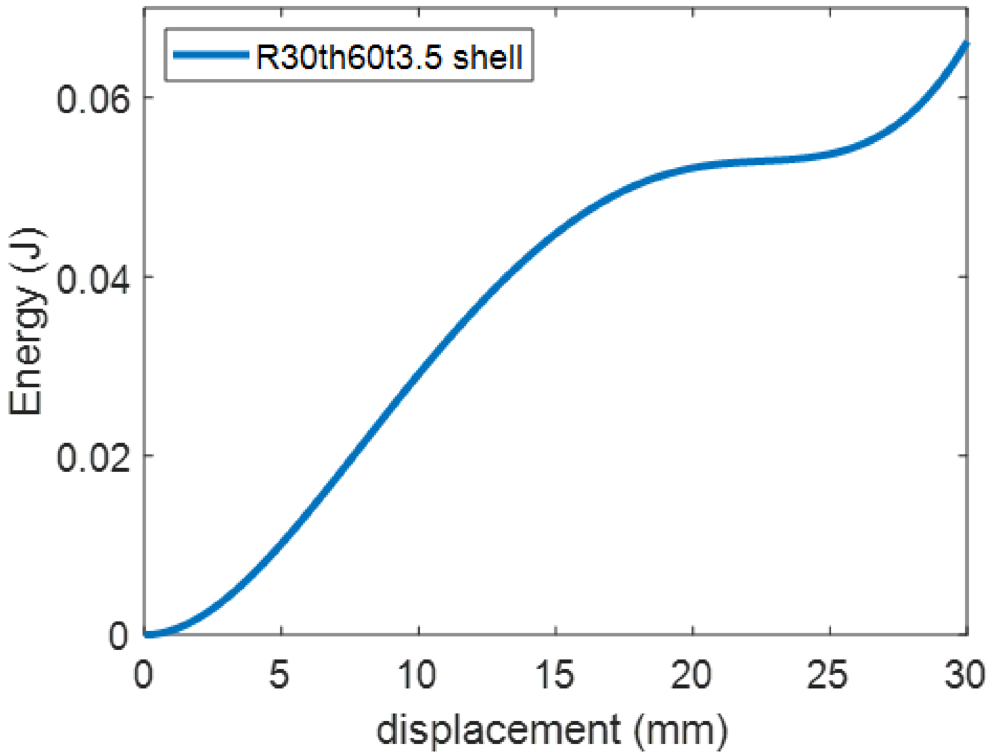

Figure S2. Strain Energy-displacement plot of selected shell for power amplification of our mechanism based on FEA simulation.

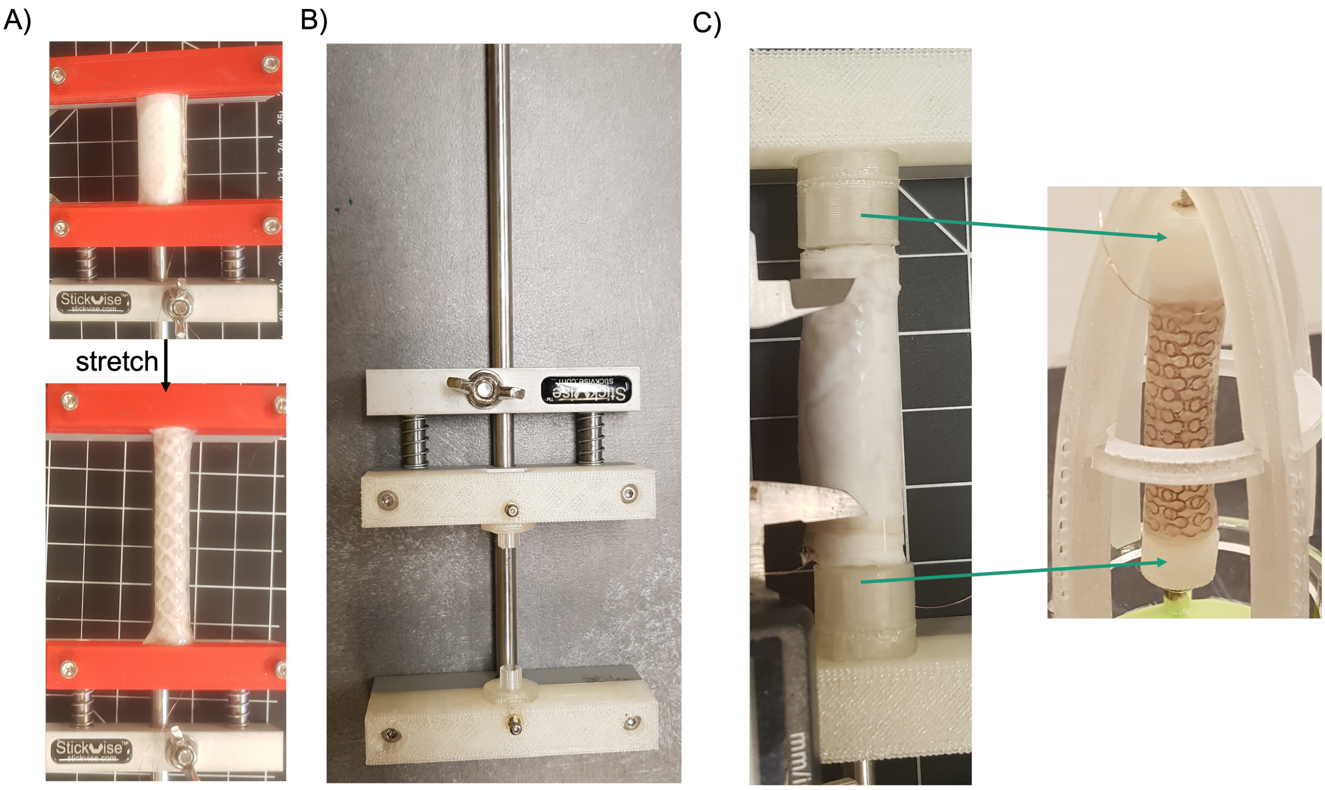

Figure S3. Modular jig and its parts used for the alignment, characterization, and assembly of the LCE actuators. A) An example of the jig with holders clamped to actuator and used for characterization. B) jig adapted to attach the rolled actuator for modular assembly. C) The inner part of the actuator with acrylic was glued to the printed part locked in the jig for the mesogen alignment step. The image on the right shows the cured actuator (in its contracted state) assembled to the robot. One end of the removable part of the jig was screwed to the top cap, and the other had a magnet glued to the other removable end of the jig for self-alignment with the magnet on the shell.

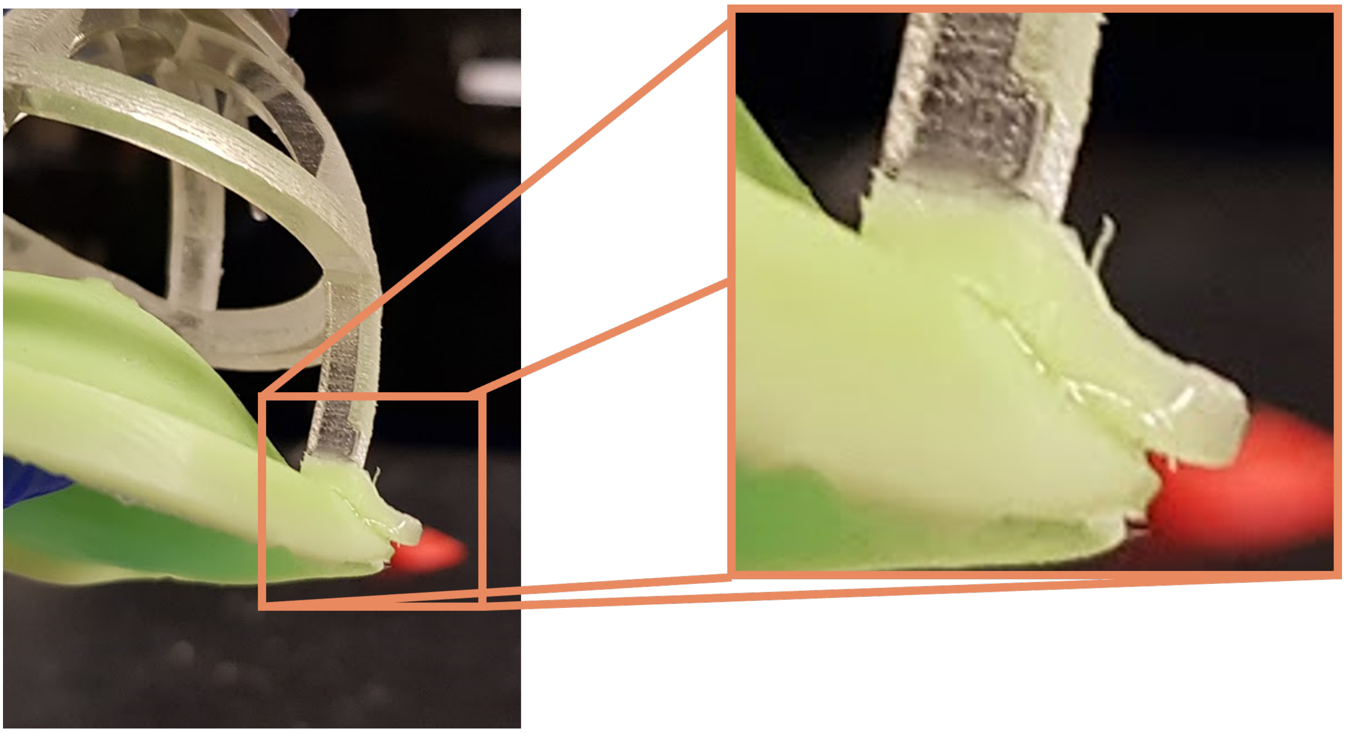

Figure S4. Demonstration of a molded soft foot and its successful bonding between a rigid layer and the shell. The boding process between the parts followed the fabrication steps as instructed by the manufacturer (Ttbonding Inc.). We applied the adhesion promoter, PolyPrep, then the activator/accelerator, heat it the surfaces once dried, and used the structural adhesive to attach the two parts. The curing was done within $\leq 20\ s$.

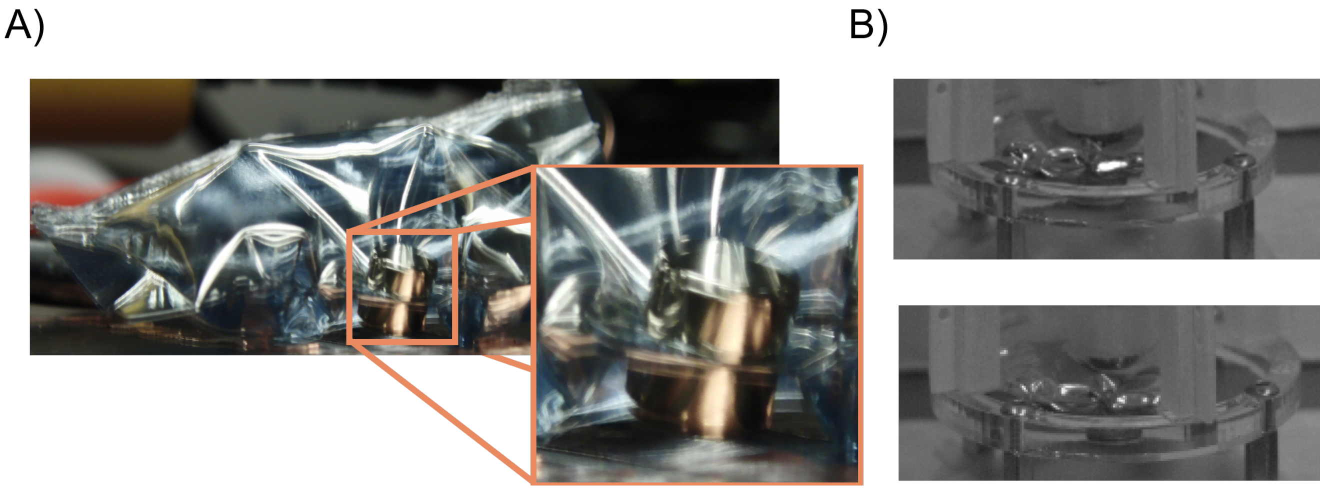

Figure S5. Detachment of magnets from the liquid-heat-activated pouch. A) Screenshot of the moment the magnets start to detach from the pouch when the low-boiling point liquid evaporates and inflate the pouch. B) Screenshot of the power amplification system demonstrating the application of the magnet with pouch to trigger the latching and decoupling of the actuator from the shell.
